# Smoking and consumption of ultra-processed foods — a combination of risky choices: A cross-sectional study using Vigitel 2018 data

**DOI:** 10.1590/1516-3180.2023.0156.R1.16022024

**Published:** 2024-07-19

**Authors:** Ana Maria Pita Ruiz, Daniela de Assumpção, Semíramis Martins Álvares Domene, Priscila Maria Stolses Bergamo Francisco

**Affiliations:** I PhD student, Collective Health Postgraduate Program, Department of Collective Health, Faculty of Medical Sciences, Universidade Estadual de Campinas (UNICAMP), Campinas (SP), Brazil.; II Professor, Gerontology Postgraduate Program, Faculty of Medical Sciences, Universidade Estadual de Campinas (UNICAMP), Campinas (SP), Brazil.; III Associate Professor, Department of Public Policies and Collective Health, Health and Society Institute, Universidade Federal de São Paulo (UNIFESP), Santos (SP), Brazil.; IV Professor, Collective Health Postgraduate Program, Department of Collective Health, Faculty of Medical Sciences, Universidade Estadual de Campinas (UNICAMP), Campinas (SP), Brazil.

**Keywords:** Tobacco use disorder, Smoking, Feeding behavior, Health surveys, Food Intake, Tobacco, Population survey, Adults

## Abstract

**BACKGROUND:**

Smoking and unhealthy diet are important risk factors for cardiovascular and metabolic diseases, contributing to public health crises.

**OBJECTIVE:**

To evaluate the consumption of natural/minimally processed and ultra-processed foods by Brazilian adults (18–59 years old) according to smoking status.

**DESIGN AND SETTING:**

Cross-sectional study of a representative population sample from 26 state capitals and the Federal District (Brazil-2018).

**METHODS:**

Data were obtained from Vigitel—Surveillance System for Risk and Protection Factors for Chronic Diseases by Telephone Survey. Participants were categorized as smokers, ex-smokers, and never smokers. Multinomial logistic regression was used for analyses.

**RESULTS:**

Of the 30,800 adults evaluated, 9.4% (95%CI: 8.7-10.2) were smokers and 16.5% (95%CI: 15.8-17.3) were ex-smokers. Smokers were less likely to consume fruit and natural juice, and more likely to consume soda or artificial juice (≥ 5 days/week) than ex-smokers and never smokers. Regarding the daily frequency of consumption, smokers were observed to be less likely to eat fruit more than 1 time/day and more likely to drink ≥ 3 cups/cans of soda/day. Compared to never smokers, smokers had a 42% higher chance of consuming ≥ 3 glasses of natural juice/day. On the day before the interview, fruit, milk, tubers, squash, and okra consumption were lower among smokers than non-smokers. Smokers were more likely to report consuming soft drinks, fruit juice, sauces, ready-made dishes, margarine, and sausages.

**CONCLUSION:**

Smokers had lower fruit consumption, and higher consumption of natural juices and ultra-processed foods. We highlight the need for strategies that encourage healthy eating and smoking cessation.

## INTRODUCTION

Smoking is responsible for the deaths of more than eight million people annually worldwide as a result of both the direct consumption of tobacco and exposure to passive smoking. More than 80% of the 1.3 billion smokers live in countries with less economic development.^1^ Regarding noncommunicable chronic diseases (NCDs), in the Americas, 16% of the deaths due to cardiovascular disease, 25% due to cancer, and 52% due to chronic respiratory diseases are attributed to tobacco use disorder.^2^ In Brazil, 28% (n = 156,337) of premature deaths in 2015 were caused by smoking, with an estimated total cost of R$ 56.9 billion in terms of health care and loss of productivity.^3^

Another important modifiable risk factor that increases the probability of developing NCDs is unhealthy eating^4^—a habit often adopted by smokers. Indeed, a study that estimated the combination of risk factors based on data from the 2015 Surveillance System for Risk and Protection Factors for Chronic Diseases by Telephone Survey (Vigitel) reported a greater likelihood among smokers of having an inadequate diet.^5^ The co-occurrence of smoking and an unhealthy diet was found in 8.6% of the adult population (18–59 years of age) residing in Brazilian state capitals and the Federal District, and 10.8% of men.^6^ In Brazil, an inadequate diet remained at the top of the list of 17 main risk factors for the global burden of disease in both sexes between 1990 and 2015, whereas smoking dropped from the second to the fourth position among men and from the fourth to the fifth position among women.^7^

In the adult population (≥ 18 years) of Belo Horizonte, the capital of the state of Minas Gerais, Gomes et al.^8^ found that the prevalence of inadequate eating habits was higher among smokers and was characterized by more frequent consumption of soda, red meat, and meat with excess fat as well as a lower consumption of fruits and vegetables. Analyzing data from three dietary records of adults (18–70 years old) from Minnesota, USA, Raatz et al.^9^ found a lower intake of energy, polyunsaturated fatty acids, dietary fiber, and micronutrients, such as calcium, iron, potassium, folate, and vitamins A, C, and E, among smokers than non-smokers. Another study found that the dietary quality scores for five of the eight indices selected were significantly diminished with smoking.^10^ Studies have also shown that smokers have an altered sense of taste, with less sensitivity to bitter, sour, and salty flavors.^11,12^

In a study of 358,218 American adults (≥ 18 years of age), Adams et al.^13^ quantified the population-attributable risk for nine chronic diseases. Among the risk factors with confirmed causality, smoking and obesity contributed to the occurrence of five to six diseases, whereas low fruit and vegetable consumption contributed to the occurrence of two. The authors also confirmed their hypothesis of a dose-response gradient between each outcome and the increase in the number of risk factors, demonstrating the additive effect of separate risk factors. Furthermore, a case-control study (2010–2015) by Fliss-Isakov et al.^14^ showed an association of high ultra-processed food intake (≥ 44.8% of total kcal) with cases of colorectal adenomas in smokers, mainly advanced (Odds ratio [OR] = 4.76) and proximal (OR = 6.23), and a significant interaction between smoking and high intake of these foods.

## OBJECTIVES

This study aimed to investigate the consumption of ultra-processed foods, and natural and minimally processed foods in Brazilian adults aged 18–59 years according to their smoking status (current smoker, ex-smoker, and never smoker). This study innovates by using questions incorporated into Vigitel in 2018, which assesses food consumption according to the NOVA classification presented in the 2014 Food Guide for the Brazilian Population.

## METHODS

### Study design and population

A cross-sectional study was conducted using data from the Surveillance System for Risk and Protection Factors for Chronic Diseases by Telephone Survey (Vigitel, in Portuguese), administered to the adult population (≥ 18 years of age) residing in 26 state capitals and the Federal District of Brazil in 2018.

The sampling procedures for the Vigitel survey sought to obtain probabilistic samples of the adult population based on records of residential telephone lines (landlines) in the state capitals and Federal District that were made available annually by the main residential telephone operators in the country. In the first stage, at least 5000 telephone lines were randomly selected from each city. This selection was systematic and stratified by the postal code. The selected lines were then divided into replicates of 200 lines each for the identification of active residential lines (eligible for the system). After confirming the eligibility of the line, the second sampling step was the selection of one of the adults (≥ 18 years of age) residing in the selected home.^15^

### Measures

In 2018, the Vigitel survey identified 73,648 eligible lines for the 26 capital cities and the Federal District, and conducted 52,395 interviews. Post-stratification weights were assigned to each interviewee to obtain reliable estimates of the adult population having a residential telephone line in each city. The weights equated the sociodemographic distribution of the Vigitel sample to the distribution estimated for the total adult population of the same city, considering the following variables: sex (female, male), age group (18–24, 25–34, 35–44, 45–54, 55–64, and ≥ 65 years) and schooling (without instruction/incomplete primary school, complete primary/incomplete high school, complete high school/incomplete higher education and complete higher education).^15^

In the present study, the questions "Do you currently smoke?" and "Did you ever smoke in the past?" were used to compose the dependent variable, with individuals categorized as smokers, ex-smokers, and non-smoker (never smoked).

The sociodemographic variables of interest were sex, age group (18 to 29, 30 to 39 and 40 to 59 years of age), schooling (0 to 8, 9 to 11 and 12 or more years of study), skin color/ethnicity (White, Black or Brown), marital status (with or without a spouse), possession of a private health insurance plan (yes or no), and region of residence (Central West, Northeast, North, Southeast, South). Individuals of yellow skin color and indigenous individuals were excluded from the study on consideration of the absolute number of respondents in the smoker category (n = 49), which impeded the acquiring of estimates with acceptable precision.

Food consumption was evaluated through questions on the weekly frequency of consumption of raw and cooked vegetables, natural fruit juices and fruits, and soda or artificial juice, which were classified as 0 to 2, 3 to 4 or ≥ 5 days (regular consumption) per week. Questions were also posed on the daily frequency of consumption of raw and cooked vegetables (once or twice per day), natural juice (1, 2 or ≥ 3 glasses), fruits (1, 2 or ≥ 3 times) and soda/artificial juice (1, 2 or ≥ 3 glasses/cans per day).

The NOVA classification of foods was used to categorize foods and beverages consumed the day prior to the interview (yes or no) according to their degree of processing:^16^

- Natural or minimally processed foods: vegetables, fruits, meats, eggs, grains, tubers, legumes, nuts, and milk;
- Ultra-processed foods: soda, fruit drinks (juice in a box or can and powdered soft drinks), sweetened milk-based drinks, sweets, chips/crisps/crackers, sauces, ready-to-eat/semi-ready-to-eat products, margarine, processed meats, sandwich bread, hotdog/hamburger buns.

### Statistical analysis

The distribution of smokers, ex-smokers, and non-smokers was determined according to sociodemographic characteristics, followed by an estimation of the proportion of food intake according to smoking status. Differences between groups were determined using Pearson’s chi-squared test with the second-order (Rao and Scott) correction, and by odds ratios (OR) adjusted for age, sex, schooling, region of residence, and alcohol abuse or binge drinking (≥ 5 drinks for men, ≥ 4 drinks for women, on a single occasion, at least once in the last 30 days). Alcohol abuse was selected as an adjustment variable owing to the high prevalence observed among smokers (43.5%; 95%CI: 39.3-47.8) and former smokers (24.0%; 95%CI: 21, 8-26.3). Multinomial logistic regression was used to estimate the ORs for food intake among smokers compared to non-smokers and ex-smokers. Associations were determined using Wald’s test, with a value of P < 0.05. Analyses were conducted using the survey module of Stata 15.1 (StataCorp LLC, College Station, Texas, United States), which considers the complex sampling of the study.

All participants received clarifications regarding the study objectives at the time of telephone contact. Written informed consent was replaced with verbal informed consent. This study was approved by the National Human Research Ethics Committee of the Health Ministry (report n. 355,590, 06/26/2013).

## RESULTS

Data from 30,800 individuals aged 18–59 years were analyzed. The mean age was 36.7 years (95%CI: 36.5-36.9). The prevalence of smokers was 9.4% (95%CI: 8.7-10.2) in the total population, 12.4% among men and 6.7% among women. The prevalence of ex-smokers was 16.5% (95%CI: 15.8-17.3) and was higher among men than women (19.1% vs. 14.2%, respectively).

Most smokers were men aged between 40 and 59 years, with less than 12 years of schooling, without a spouse, without a private health insurance plan, and resided in the southeastern region of the country (P < 0.0001). Similar results were found for ex-smokers, except that the majority had a spouse (Table 1).

**Table 1:** Sociodemographic characteristics of adults residing in Brazilian state capitals and the Federal District according to smoking status. Vigitel, Brazil, 2018

Variable		% (95% CI)			P value*
		Never smoked	Ex-smoker	Smoker	
Sex					
	Male	43.54 (42.29-44.80)	54.45 (51.86-57.00)	62.03 (57.99-65.90)	**< 0.0001**
	Female	56.46 (55.20-57.71)	45.55 (43.00-48.14)	37.97 (34.10-42.01)	
Age group (in years)					
	18 to 29	35.40 (34.17-36.65)	19.32 (17.27-21.55)	25.49 (21.73-29.65)	**< 0.0001**
	30 to 39	27.82 (26.67-29.00)	22.77 (20.53-25.18)	26.55 (22.50-31.03)	
	40 to 59	36.78 (35.67-37.91)	57.91 (55.27-60.50)	47.96 (43.77-52.18)	
Schooling (years of study)					
	0 to 8	17.63 (16.62-18.68)	37.43 (34.85-40.07)	39.36 (35.25-43.62)	**< 0.0001**
	9 to 11	43.26 (42.05-44.48)	37.21 (34.80-39.69)	38.72 (34.53-43.08)	
	12 or more	39.11 (37.91-40.33)	25.36 (23.26-27.59)	21.92 (18.95-25.22)	
Ethnicity/skin color					
	White	43.28 (42.00-44.56)	43.85 (41.19-46.54)	44.61 (40.56-48.74)	0.2596
	Black	12.22 (11.35-13.16)	9.94 (8.45-11.66)	12.15 (9.59-15.28)	
	Brown	44.50 (43.25-45.75)	46.21 (43.54-48.90)	43.24 (39.12-47.45)	
Marital status					
	Without spouse	56.72 (55.49-57.93)	43.63 (41.10-46.20)	59.99 (55.86-63.97)	**< 0.0001**
	With spouse	43.28 (42.07-44.51)	56.37 (53.80-58.90)	40.01 (36.03-44.14)	
Health insurance					
	No	51.65 (50.41-52.89)	60.72 (58.19-63.20)	67.69 (63.79-71.36)	**< 0.0001**
	Yes	48.35 (47.11-49.59)	39.28 (36.80-41.81)	32.31 (28.64-36.21)	
Region of residence					
	Central West	12.36 (11.79-12.95)	11.26 (10.13-12.51)	11.28 (9.69-13.09)	**< 0.0001**
	Northeast	27.63 (26.75-28.53)	23.69 (22.00-25.47)	16.56 (14.47-18.88)	
	North	11.09 (10.59-11.61)	12.23 (10.96-13.62)	7.48 (6.17-9.04)	
	Southeast	41.82 (40.46-43.18)	43.68 (40.93-46.46)	53.79 (49.72-57.82)	
	South	7.10 (6.68-7.55)	9.14 (8.17-10.2)	10.90 (9.31-12.72)	

* P value from Pearson’s chi-square test (Rao-Scott). CI = confidence interval.

Regarding the weekly frequency of consumption (number of days in the week), compared to non-smokers, smokers were less likely to regularly consume (five or more days/week) natural fruit juice and fruits, and more likely to consume soda or artificial juice. The same pattern was observed in smokers compared to ex-smokers (Table 2).

**Table 2:** Weekly frequency and odds ratios of food intake in adults residing in Brazilian state capitals and the Federal District according to smoking status. Vigitel, Brazil, 2018

Weekly food frequency		Never smoked (0)	Ex-smoker (1)	Smoker (2)	OR^a^ (2/1)	P^b^	OR^a^ (2/0)	P^b^
Raw vegetables		P = 0.1644^c^						
	< 5 times	60.81	60.75	64.87	1		1	
	≥ 5 times	39.19	39.25	35.13	0.87	0.254	1.02	0.848
Cooked vegetables		**P = 0.0001** c						
	< 5 times	73.88	77.63	80.18	1		1	
	≥ 5 times	26.12	22.37	19.82	0.93	0.520	0.87	0.210
Natural juice		**P < 0.0001** c						
	< 5 times	70.08	74.60	78.79	1		1	
	≥ 5 times	29.92	25.40	21.21	0.80	**0.046**	0.69	**< 0.001**
Fruits		**P < 0.0001** c						
	< 5 times	52.70	52.18	71.06	1		1	
	≥ 5 times	47.30	47.82	28.94	0.49	**< 0.001**	0.51	**< 0.001**
Soda/artificial juice		**P < 0.0001** c						
	< 3 times	73.14	73.08	57.27	1		1	
	≥ 3 times	26.86	26.92	42.73	1.72	**< 0.001**	1.90	**< 0.001**

a Odds ratio (OR) controlled for age, sex, schooling, region of residence, and alcohol abuse

b P value from Wald’s test

c P value from Pearson’s chi-square test (Rao-Scott).

In the analyses about the daily (number of times a day) frequency of consumption, the smokers were less likely to consume fruits more than once per day and more likely to ingest three or more glasses/cans of soda per day than non-smokers and ex-smokers. Paradoxically, smokers were 42% more likely than non-smokers to consume three or more glasses of natural fruit juice per day (Table 3).

**Table 3:** Frequency and odds ratios of daily food intake in adults residing in Brazilian state capitals and the Federal District according to smoking status. Vigitel, Brazil, 2018

Daily food intake		Never smoked (0)	Ex-smoker (1)	Smoker (2)	OR^a^ (2/1)	P^b^	OR^a^ (2/0)	P^b^
Raw vegetables		P = 0.7487^c^						
	1 time	72.67	73.80	72.93	1		1	
	2 times	27.33	26.20	27.07	1.05	0.659	1.02	0.854
Cooked vegetables		P = 0.7826^c^						
	1 time	67.10	66.02	66.37	1		1	
	2 times	32.90	33.98	33.63	0.96	0.754	1.04	0.720
Natural juice		**P = 0.0087** c						
	1 glass	42.97	40.97	35.28	1		1	
	2 glasses	35.47	35.29	36.26	1.10	0.481	1.18	0.174
	≥ 3 glasses	21.56	23.74	28.47	1.21	0.252	1.42	**0.028**
Fruits		**P = 0.0001** c						
	1	47.65	49.63	57.52	1		1	
	2	35.04	31.56	27.46	0.77	**0.043**	0.67	**0.001**
	≥ 3 times	17.31	18.81	15.03	0.72	**0.031**	0.70	**0.012**
Soda/artificial juice		**P < 0.0001** c						
	1 glass/can	42.13	38.64	30.59	1		1	
	2 glasses/cans	37.20	35.37	33.30	1.12	0.438	1.13	0.355
	≥ 3 glasses/cans	20.67	25.99	36.10	1.46	**0.022**	2.00	**< 0.001**

a Odds ratio (OR) controlled for age, sex, schooling, region of residence, and alcohol abuse

b P value from Wald’s test.

c P value from Pearson’s chi-square test (Rao-Scott).

Regarding natural and minimally processed foods, smokers were less likely to have consumed fruits and milk on the day prior to the interview compared to non-smokers and ex-smokers, and less likely to have consumed tubers and vegetables, such as pumpkin and okra, compared to non-smokers (Table 4). Regarding ultra-processed foods consumed the day prior to the interview, smokers were more likely to have consumed soda, fruit drinks, sauces, ready-to-eat, or semi-ready-to-eat products, margarine, and processed meats than ex-smokers and non-smokers (Table 5).

**Table 4:** Frequency and odds ratios of consumption of natural/minimally processed foods on the day prior to the interview among adults residing in Brazilian state capitals and the Federal District according to smoking status. Vigitel, Brazil, 2018

Food groups		Never smoked (0)	Ex-smoker (1)	Smoker (2)	OR^a^ (2/1)	P^b^	OR^a^ (2/0)	P^b^
Natural or minimally processed foods								
Vegetables		P = 0.0562^c^						
	No	21.10	22.72	25.13	1		1	
	Yes	78.90	77.28	74.87	0.92	0.491	0.94	0.583
Fruits		**P < 0.0001** c						
	No	21.94	23.74	37.68	1		1	
	Yes	78.06	76.26	62.32	0.57	**< 0.001**	0.53	**< 0.001**
Milk		**P = 0.0001** c						
	No	43.68	43.82	52.56	1		1	
	Yes	56.32	56.18	47.44	0.77	**0.011**	0.77	**0.006**
Legumes		P = 0.9330^c^						
	No	26.87	26.49	26.38	1		1	
	Yes	73.13	73.51	73.62	0.98	0.878	0.93	0.444
Grains		P = 0.1368^c^						
	No	13.82	16.11	14.67				
	Yes	86.18	83.89	85.33	1.06	0.681	0.83	0.139
Meats		P = 0.7443^c^						
	No	10.26	10.46	11.27	1		1	
	Yes	89.74	89.54	88.73	0.94	0.712	0.93	0.647
Eggs		P = 0.5870^c^						
	No	58.53	58.12	56.44	1		1	
	Yes	41.47	41.88	43.56	1.05	0.619	1.08	0.402
Tubers, pumpkin or okra		**P < 0.0001** c						
	No	36.32	42.46	44.02	1		1	
	Yes	63.68	57.54	55.98	0.98	0.859	0.81	**0.025**
Nuts		P = 0.0833^c^						
	No	83.85	83.98	87.04	1		1	
	Yes	16.15	16.02	12.96	0.81	0.117	0.84	0.149

a Odds ratio (OR) controlled for age, sex, schooling, region of residence, and alcohol abuse

b P value from Wald’s test

c P value from Pearson’s chi-square test (Rao-Scott).

**Table 5:** Frequency and odds ratios of consumption of ultra-processed foods on the day prior to the interview among adults residing in Brazilian state capitals and the Federal District according to smoking status. Vigitel, Brazil, 2018

Food groups		Never smoked (0)	Ex-smoker (1)	Smoker (2)	OR^a^ (2/1)	P^b^	OR^a^ (2/0)	P^b^
Ultra-processed foods								
Soda		**P < 0.0001** c						
	No	73.40	73.49	57.96	1		1	
	Yes	26.60	26.51	42.04	1.77	**< 0.001**	2.00	**< 0.001**
Other sweetened beverages		**P < 0.0001** c						
	No	75.16	72.79	66.04	1		1	
	Yes	24.84	27.21	33.96	1.26	**0.047**	1.46	**< 0.001**
Sweetened milk-based beverages		**P = 0.0020** c						
	No	72.55	75.48	78.52	1		1	
	Yes	27.45	24.52	21.48	0.82	0.118	0.87	0.220
Chips/crisps/crackers		P = 0.4035^c^						
	No	77.63	76.97	75.20	1		1	
	Yes	22.37	23.03	24.80	1.01	0.905	1.02	0.866
Sauces/ready-semi-ready-to-eat products		**P < 0.0001** c						
	No	77.71	76.82	69.21	1		1	
	Yes	22.29	23.18	30.79	1.27	**0.046**	1.54	**< 0.001**
Margarine		**P = 0.0020** c						
	No	55.63	55.33	48.29	1		1	
	Yes	44.37	44.67	51.71	1.27	**0.019**	1.23	**0.027**
Sweets		P = 0.1890^c^						
	No	56.87	59.80	58.52	1		1	
	Yes	43.13	40.20	41.48	1.01	0.904	1.09	0.387
Processed meats		**P < 0.0001** c						
	No	74.17	71.01	62.11	1		1	
	Yes	25.83	28.99	37.89	1.31	**0.017**	1.63	**< 0.001**
Sandwich bread/hotdog or hamburger buns		P = 0.9532^c^						
	No	64.50	64.56	65.11	1		1	
	Yes	35.50	35.44	34.89	0.89	0.326	1.00	0.963

a Odds ratio (OR) controlled for age, sex, schooling, region of residence, and alcohol abuse

b P value from Wald’s test

c P value from Pearson’s chi-square test (Rao-Scott).

## DISCUSSION

The results of the present study enabled identification of the dietary habits of smokers in comparison to those of ex-smokers and individuals who have never smoked. Smoking and a dietary pattern marked by high consumption of ultra-processed foods are among the modifiable risk factors that have an evident impact on health outcomes. Smokers had lower fruit intake and greater consumption of soda or artificial juice in both the weekly and daily frequency analysis. Daily consumption of three or more glasses of natural juice was higher among smokers than among non-smokers. On the day prior to the interview, smokers showed lower consumption of fruits, milk, and tubers, as well as greater consumption of ultra-processed foods such as soda, fruit drinks, sauces, ready-to-eat products, margarine, and processed meats.

The prevalence of smokers and ex-smokers in the population aged between 18 and 59 years residing in the Brazilian state capitals and Federal District was 9.4% and 16.5%, respectively. Data from the Vigitel survey on the trend of smoking indicators in adults (18 years of age or older) reveal a reduction in the prevalence of smokers (from 15.6% to 10.8%) and ex-smokers (22.2% to 21.2%) from 2006 to 2014.^17^ According to the National Health Survey (Pesquisa Nacional de Saúde - PNS), the prevalence of all tobacco use indicators diminished between 2013 and 2019, and the prevalence of ex-smokers increased from 17.5% to 26.6% (prevalence ratio: 1.52; 95%CI: 1.46-1.58).^18^ Brazil is recognized for combating tobacco use. In 2005, with the issuance of the guidelines of the World Health Organization Framework Convention on Tobacco Control, which consolidated the National Tobacco Control Policy, Brazil successfully implemented anti-smoking measures, such as the prohibition of advertising for tobacco products, an increase in the price of cigarettes, the use of warning images on cigarette packs, and the banning of smoking in closed group environments.^19,20^

The prevalence of smokers was higher among men, individuals aged 40–59 years, those without a spouse, those with a lower level of schooling, those who did not possess a private health insurance plan, and those who resided in the southeastern region of Brazil. Similar findings have been reported in previous studies.^17,21^ The 2019 National Health Survey also found that tobacco use was greater among men, individuals between 40 and 59 years of age, those with less schooling and a low income, Black and Brown individuals, and residents of the southern, central western, and southeastern regions.^18^ Using data from the 2008–2009 Family Budget Survey (Pesquisa de Orçamentos Familiares - POF), Bazotti et al.^21^ found that approximately 10% of the Brazilian population reported spending on tobacco products, substantially impacting the family budget.

Smokers reported lower consumption of fruits and natural fruit juices, and greater consumption of soda and artificial juice. Using data from the 2015 Vigitel survey, Francisco et al.^5^ found that smokers had a greater likelihood (OR = 1.50; 95%CI: 1.20-1.87) of having an inadequate diet, which was evaluated using an indicator comprising foods considered to be protectors from chronic disease (fruits, raw and cooked vegetables, milk and beans) and risk foods (sweets, red meat, soda and other sweetened beverages). In the city of Belo Horizonte, Brazil, 24.8% of non-smokers and 36.9% of smokers had an unhealthy diet (OR = 1.82; 95%CI: 1.49-2.22) characterized by the frequent consumption of soda or artificial juice, red meat, and meat with excess fat as well less frequent consumption of fruits and vegetables.^8^ Notably, no studies were found in the literature that explore the consumption of specific foods and beverages according to smoking status. The present investigation is also the first to determine a greater likelihood of consumption of ultra-processed foods among smokers.

Some studies have investigated nutrient intake and overall diet quality according to the smoking habit. Raatz et al.^9^ compared the usual nutrient intake of smokers and non-smokers aged 18–70 years in the United States and found a lower intake of energy, polyunsaturated fatty acids, linoleic acid, dietary fiber, calcium, iron, magnesium, potassium, phosphorus, vitamins A, C and E, riboflavin, niacin, pantothenic acid, pyridoxin and folate among smokers. In addition, studies have shown that smokers have higher levels of oxidative stress, which increases the importance of consuming healthy antioxidant foods.^22,23^

A population-based cross-sectional study conducted in Luxemburg with individuals aged 18–64 years detected a significant inverse association between overall diet quality and smoking intensity, measured by the quantity of cigarettes smoked per day. Lower diet quality scores were found for five of the eight indices evaluated in the study, revealing poorer diet quality among moderate and heavy smokers compared to non-smokers after controlling for age, sex, schooling, cardiovascular health indicators, physical activity, adiposity, and alcohol intake.^10^ Compared to those who had never smoked, ex-smokers had lower intake of processed meats, refined grains, solid fats, added sugars, and alcohol.^10^ In a study conducted in the city of São Paulo, Brazil, Andrade et al.^24^ found poorer dietary quality in smokers based on an indicator composed of 12 components (nine food groups, two nutrients, and a component corresponding to the percentage of energy obtained from solid fats, alcohol, and added sugars).

The consumption of three or more glasses of natural fruit juice per day by smokers merits further attention. Evidence from a systematic review and meta-analysis indicated a statistically significant association between the consumption of fruit juice (without added sugars) and a greater risk of weight gain and insulin resistance detected using the HOMA-IR index.^25^ Furthermore, international guidelines establish a maximum of 240 ml/day for adults.^26^ Data from the individual food intake module of the 2008–2009 Family Budgets Survey revealed that 5.49% of total energy from the diet of the Brazilian population came from fruits, with an important part of this energy derived from juices (2.45%).^27^ The consumption of fruit juices does not offer the same health benefits as consuming the whole fruit, since the extraction process reduces the fiber content, leads to loss of nutrients that are sensitive to light, oxygen, and heat, and generates a lower sensation of satiety.^27,28^

Robust evidence from a set of observational studies indicate a direct association between the consumption of ultra-processed foods and adverse health outcomes.^29^ A randomized clinical trial showed that a diet rich in ultra-processed foods promotes gain in body weight.^30^ The concomitance of smoking and consumption of ultra-processed foods is a risky combination, especially among men with a low level of schooling. These findings are consistent with those of other studies on poor dietary quality in this population.

To our knowledge, no existing study has specifically analyzed the consumption of different foods according to smoking status (smokers, ex-smokers, and non-smokers). Thus, the present results can contribute to future studies along this line. The Vigitel telephone survey involves a variety of questions on the frequency of consumption of foods considered markers of healthy and unhealthy diets, and on the use of cigarettes. Among the limitations related to the methods adopted by the Vigitel survey, the information reported by the participants regarding food consumption may have been subject to recall bias. Therefore, it was impossible to establish causal relationships because of the cross-sectional design of the study. Moreover, the sample of the Vigitel survey is restricted to individuals with a residential telephone line (landline) who reside in state capitals and the Federal District, which limits the representativeness of the findings. However, the use of weighting factors reduced this problem by equating the demographic characteristics of the sample with those of the adult population in Brazil.

## CONCLUSION

The present study enabled us to outline the food consumption profile of smokers, who were found to have a lower weekly and daily frequency of fruit consumption and a greater frequency of soda or artificial juices consumption compared to ex-smokers and non-smokers. Attention is to be drawn to the high consumption of natural fruit juices (three or more glasses per day) among smokers. On the day prior to the interview, smokers reported a lower frequency of consumption of natural and minimally processed foods, such as fruits, milk, and tubers, as well as a greater frequency of ultra-processed foods, such as soda, fruit drinks, sauces, ready-to-eat products, margarine, and processed meats. These findings underscore the importance of promoting a healthy diet, especially among smokers, through strategies that consider concomitance of these risk behaviors.
